# An evaluation of adherence to folic acid supplementation in pregnant women during early gestation for the prevention of neural tube defects

**DOI:** 10.1017/S1368980022001574

**Published:** 2022-11

**Authors:** Anna Linnell, Niamh Murphy, Jon Godwin, Alexandra Cremona

**Affiliations:** 1 School of Allied Health (SAH), University of Limerick, Limerick, Ireland; 2 Irish Nutrition and Dietetic Institute (INDI), Dublin, Ireland; 3 Department of Dietetics, University Maternity Hospital Limerick, Limerick, Ireland; 4 Maternity Dietetics Ireland (MDI), Dublin, Ireland; 5 Nuffield Department of Population Health, Medical Sciences Division, University of Oxford, England, UK; 6 Health Research Institute, School of Allied Health, Faculty of Education and Health Sciences, University of Limerick, Limerick, Ireland

**Keywords:** Neural tube defects, Folic acid supplementation, Folic acid, Obesity pregnancy

## Abstract

**Objective:**

Neural tube defects (NTD) are potentially preventable by periconceptual folic acid supplementation. Women with obesity are at higher risk of NTD, therefore, are recommended a higher dose of 5 mg folic acid to mitigate this risk. The aim of this study was to evaluate maternal practice of folic acid supplementation amongst the antenatal population in relation to maternal obesity status.

**Design:**

Prospective observational study.

**Setting:**

Women ≤18 weeks’ gestation at their first antenatal appointment attending University Maternity Hospital Limerick (Ireland) were recruited. Maternal height and weight were measured. Obesity was defined at a threshold of ≥30·0 kg/m^2^ and ≥27·5 kg/m^2^ when adjusting for ethnicity. A two-part questionnaire captured maternal characteristics and assessed supplementation compliance, commencement and dosage. Fisher’s exact test for independence analysed differences in variables. A *P* value of <0·05 was considered significant.

**Participants:**

A total of 328 women participated over a duration of 6 weeks.

**Results:**

Mean gestational age was 12·4 ± 1·4 weeks and mean BMI 26·7 kg/m^2^ ± 5·2 kg/m^2^. 23·8 % (*n* 78) were classified as obese. 96·5 % (*n* 315) were taking folic acid and 95·7 % (*n* 314) supplemented daily. 30·2 % (*n* 99) commenced supplementation 12 weeks prior to conception. Overall, 57·9 % (*n* 190) of women met folic acid supplementation dose requirements. 89·1 % (*n* 55) of women with obesity did not. Women with obesity were less likely to meet the higher folic acid supplementation dose requirements (*P =<* 0·001).

**Conclusion:**

Folic acid supplementation practices within this cohort were suboptimal to prevent their risk of NTD. This study showed inadequate compliance of folic acid supplementation, and inadequate dosage for women with obesity. Increased patient education and awareness are needed within the antenatal period of pregnancy to bring folic acid supplementation practices in line with best practice guidelines.

Neural tube defects (NTD) are complex congenital malformations resulting from the incomplete closure of the neural tube during embryogenesis between 21 and 28 d after conception^(1,2)^. The failure of proper neural tube closure results in NTD involving the brain, spinal cord, meninges, skull and spine^(3)^. These defects include spina bifida, anencephaly, encephalocele and iniencephaly^(2)^. Collectively, NTD carry a heavy burden on illness with the severity of the defect ranging from mild to severe and potentially leading to death^(4)^. The two most common NTD include anencephaly, which is incompatible with life, and spina bifida, which has increased perinatal and infant mortality rates^(5)^. Although 80 % of infants with spina bifida survive the condition, it is still associated with varying degrees of physical disability^(5,6)^. Consequently, NTD can carry both short- and long-term difficulties not only for the pregnant woman but also for their offspring and family^(7)^. Economically, NTD are also associated with high indirect and direct costs for both the individual and healthcare services^(8)^.

Given the known adverse effects, it is alarming to note that Irish rates of NTD are amongst the highest in Europe when comparing rates from 1981 to 2015^(9)^. A recent study by McDonnell *et al.* (2018) demonstrated that there has been no decrease in Irish NTD occurrence as incidence rates between 2012 and 2015 were 1·05 per 1000 births, which was remarkably similar to the rate of 1·04 per 1000 births reported in the previous 3 year period^(9)^. While the rate in Ireland remains above of the rest of Europe as a whole, the rate in all the regions of the UK is similar to that of Ireland^(2)^. In contrast, NTD rates in Ireland are significantly higher than in countries where mandatory food fortifications are in place, such as USA and South America^(2)^. Since the implementation of the mandatory folic acid food fortification policy data from these countries has shown a significant reduction in the number of NTD, ranging from 35 % of births affected by NTD in the USA (63) and 50–78 % in Canada (2). In these surveillance studies, they found that the areas with the highest incidence rate observed the greatest reduction of the numbers of pregnancies affected by NTD. The consumption patterns of fortified foods and the blood folate status of the population prior to the introduction of mandatory fortification were also thought to be responsible for the high proportion of reduction of NTD observed in these countries (2,63–64).

Research has linked high rates of NTD with low folate status resulting from inadequate folate intake^(4,10,11)^. Dietary sources alone have been insufficient to confer protection as voluntary folic acid fortification strategies that are currently in place have failed to improve the levels of NTD in Europe over the past 25 years^(2,12,13)^. In addition, Whitehead *et al.* (1995) indicated for the Irish population to have a genetic background that is vulnerable to neural tube birth defects, making this an even more important opportunity for prevention of NTD through folic acid supplementation^(14)^. Therefore, the achievement of optimal folate status is an important public health goal for Ireland as it is for other populations worldwide. Hence, there is increased reliance on supplemental folic acid to optimise folate status in the preconception period in women today.

As evidenced from two landmark randomised controlled trials in the early nineties, periconceptual folic acid supplementation can prevent >70 % of NTD^(10,11)^. Subsequently, national guidelines published since 1992 recommend periconceptual folic acid supplementation to prevent both the occurrence and reoccurrence of NTD^(13,15)^. Adequate folic acid through supplementation supports the effective closure of the neural tube by optimizing red blood cell folate levels^(10,11)^. The WHO recommends that RBC folate concentrations should be above 906 nmols/l in women of reproductive age to reduce the risk of NTD^(16)^. In order to achieve this desired RBC folate level, the duration and dose of folic acid supplementation also need to be considered^(17)^. Evidence indicates that an average of 12 weeks supplementation with 400 μg folic acid in addition to dietary intake of folate-rich foods is sufficient to reach this required RBC folate level^(17–20)^. Guidelines recommend that women of childbearing age should supplement with 400 μg daily in addition to dietary sources for at least 12 weeks prior to conception^(17,18,21)^. More recent clinical guidelines recommend for such supplemental practice to be continued throughout pregnancy and into lactation^(21)^. This is due to the positive long-term impact that the adequate maternal folate status can have on the development of the fetus *in utero* and the support it can provide throughout pregnancy and lactation^(9,22–25)^.

Maternal obesity is associated with an increased risk of NTD^(26)^. Supplementation of folic acid is a highly effective means to optimise folate status. Based on a BMI of ≥30·0 kg/m^2^ and a ‘modified’ threshold of ≥27·5 kg/m^2^ for those of an Asian descent, maternal obesity has become a growing clinical priority in obstetrics today^(27,28)^. The prevalence of maternal obesity is high and rising nationally and globally^(29,30)^. Fattah *et al.* (2010) reported 19 % of women to present with obesity in their first trimester of pregnancy in a recent prospective study carried out in Dublin, Ireland^(31)^. This was the same in Limerick^(32)^ and higher (25 %) in Galway^(33)^. Such levels of obesity have also been found in Britain and in the USA^(34,35)^. Hence, it may be questioned if such obesity rates are related to the growing NTD levels today as maternal obesity and severe obesity are associated with a 1·7-fold and >3-fold increased risk of NTD, respectively^(36)^.

Possible mechanisms for this known relationship originate from the link between obesity with altered glucose metabolism^(36)^. For example, gestational diabetes mellitus has been shown to increase the risk of birth defects and can be found more frequently in women with obesity^(32,37)^. In fact, those with a higher pre-pregnancy BMI have an even greater risk of gestational diabetes mellitus^(37,38)^. Additionally, women with obesity are prone to hyperinsulinaemia, which is independently associated with having an NTD-affected pregnancy^(39,40)^. Other explanations suggest that the link is based on the altered response women with obesity have for critical nutrients^(36)^. As evidenced by O’Malley *et al.* (2018) and Werler *et al.* (1996), women with obesity have lower serum folate levels and are less responsive to the standard 400 μg folic acid required to prevent NTD in comparison with those of normal weight^(26,41)^. Thus, due to these known pregnancy complications, women with obesity are prescribed a higher dose of 5 mg folic acid to mitigate the risk of NTD^(21)^. This 5 mg dose is advised to be taken pre-conceptually and should be continued throughout the first trimester of pregnancy^(21,27)^. Following this, women with obesity are recommended to supplement daily with 400 μg folic acid as a single supplement or with a pregnancy-specific multivitamin for the remaining duration of pregnancy and breastfeeding^(2,4,21)^.

The purpose of this study was to describe folic acid supplementation practices amongst women in early pregnancy attending their first antenatal appointment at the University Maternity Hospital Limerick, Ireland. Supplementation practices were observed in relation to dose, time of supplementation and duration. In addition, we set out to understand if women with obesity were meeting the required higher dose of folic acid supplementation.

## Methods

**Fig. 1:**
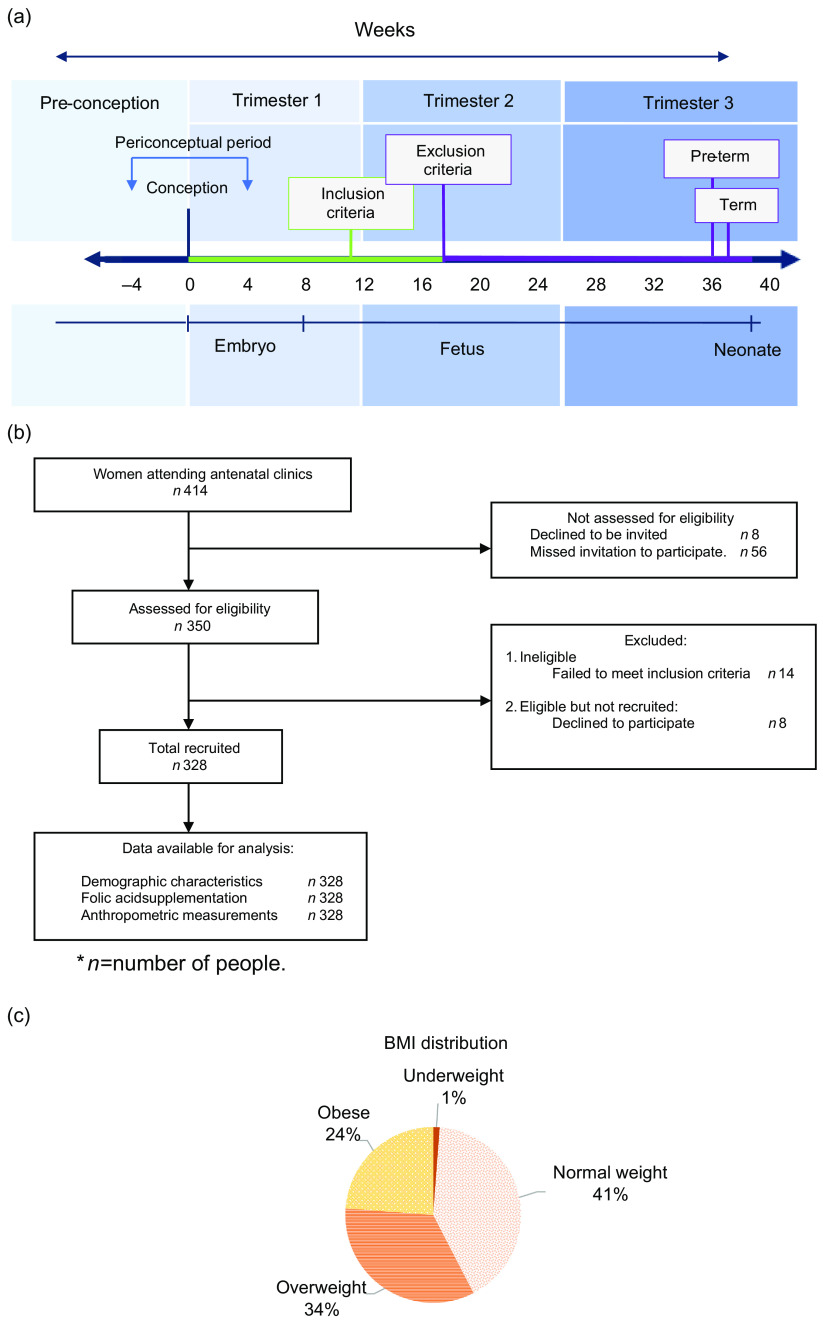
(a) Pregnancy timeline highlighting inclusion and exclusion criteria. (b) Participant recruitment classification. (c) BMI distribution. *n = number of people

A 6-week prospective observational study was carried out within the University Maternity Hospital Limerick. The inclusion criteria included women who were ≤18 weeks’ gestation, attending their first antenatal appointment with sonographic confirmation of an ongoing pregnancy. Figure 1(a) puts this criteria into context in terms of the duration of pregnancy. Women who were aged <18 years and who were unable to provide consent were excluded and not recruited for the purpose of this study.

Women were recruited at their first antenatal visit. Each participant was approached by the researcher. If the women agreed to participate, the relevant information sheet and consent form were provided. Following written informed consent, the relevant questionnaire was given (see online Supplemental Material 1), completed independently and returned to the researcher. Anthropometric measurements and relevant calculations were then performed.

### Questionnaire

A two-part questionnaire captured maternal characteristics and folic acid supplementation practices. Maternal characteristics included age, ethnicity, employment status, education level, smoking, alcohol status, gestation and parity. In addition, folic acid supplementation practices including compliance, commencement, folic acid brand, folic acid supplementation dosage and reason for choice were also investigated and compared with the Health Service Executive (HSE) (2019) folic acid supplementation guidelines. Folic acid dose was identified from assessing the brand specified and/or the dose reported by women within the questionnaire.

### Anthropometric measurements

Participants anthropometric measurements were performed by the researcher consistent with the international anthropometric standards devised by the International Society for the Advancement of Kinanthropometry^(42)^.

Height was measured to the nearest 0·1 cm using a SECA stadiometer with women standing straight, bare feet together with heals, buttocks and upper back touching the metre stick. Weight was measured digitally using a SECA electronic weighing scales to the nearest 0·1 kg with women wearing light clothing and no shoes. BMI was then calculated and classified in accordance to the WHO BMI (kg/m^2^) categories where underweight was classified at a BMI of <18·5 kg/m^2^, normal weight: 18·5–24·4 kg/m^2^, overweight: 25·0–29·9 kg/m^2^ and obesity was classified at a threshold of ≥30·0 kg/m^2^ for^(43)^. When controlling for ethnicity, a modified threshold of ≥27·5 kg/m^2^ was used to classify obesity for those identifying as of Asian descent^(28)^.

### Statistical approach

Participants’ anthropometric measurements and questionnaire results were anonymised and coded within a Microsoft Excel spreadsheet. Continuous variables were collapsed into categories where appropriate. Data were exported and analysed using IBM (2020) Statistical Package for Social Sciences (SPSS) V26.0. The distributions of continuous variables were first investigated for normality using the Kolmogorov–Smirnov test. Descriptive statistics were then used to describe the characteristics of the study cohort. *χ*^2^ test using cross-tabulation was used to assess the relationship between being obese and non-obese and reaching folic acid supplementation requirements. For such analysis, women with a BMI categorised as underweight, normal-weight and overweight were grouped as ‘not obese’. Those with a BMI classified as obese remained within the ‘obese’ group. Fisher’s exact tests for independence were then used to analyse differences in variables where low numbers rendered the *χ*^2^ tests invalid. A *P* value of <0·05 was considered significant for all analyses.

## Results

### Maternal characteristics

A total of 350 attended their first antenatal booking appointment over 6 weeks, whereby 328 women were recruited for this study. Overall, fourteen women were excluded as they failed to meet this studies inclusion criteria, sixty-four failed to attend on the given days and eight women declined to participate as represented in Fig. 1(b).

Maternal characteristics were assessed as seen in Table 1. From women’s self-reported ethnicity, it was seen that this cohort consisted predominately of Caucasians (91·2 %). Those of an Asian-descent represented a small minority of 7·0 %. Additionally, a high percentage of women (57·3 %) reported to be educated to third level and to be working full time (54·6 %). Over half of women stated that they were not smoking and drinking at present.

**Table 1:** Maternal characteristics

	*n*	%
Age (years)		
Median	32	
IQR	28,35	
Weight (kg)		
Mean	72·0	
sd	14·2	
BMI (kg/m^2^)		
Mean	26·7	
sd	5·2	
Ethnicity		
Caucasian	299	91·2
Asian	23	7·0
American	3	0·9
African	2	0·6
Australian	1	0·3
Highest educational qualification		
Primary/secondary	66	20·1
Vocational/training programme	56	17·1
Third level/postgraduate level	188	57·3
None of the above	9	2·7
No answer	9	2·7
Current employment status		
Full time	179	54·6
Part time	56	17·1
Unemployed	78	23·8
Student	8	2·4
No answer	7	2·1
Smoking status		
Yes	42	12·8
No	203	61·9
Stopped since I found out that I was pregnant	83	25·3
Alcohol consumption status		
Yes, drinking alcohol	2	0·6
No, I am not drinking alcohol	207	63·1
Stopped since I found out I was pregnant	119	36·3

IQR, interquartile range.

From assessing BMI as per Fig. 1(c), it was seen that 1·2 % (*n* 4) were recorded as underweight, 41·2 % (*n* 135) as normal weight, 33·8 % (*n* 111) as overweight and 23·8 % (*n* 78) as obese. Among the non-Asian population (*n* 305), it was subdivided where 1·0 % (*n* 3) had an underweight BMI, 42·0 % (*n* 128) normal-weight BMI, 33·1 % (*n* 101) overweight BMI and 23·9 % (*n* 73) had an obese BMI. For those identifying as Asian (*n* 23), 4·3 % (*n* 1) were classed as having an underweight BMI, 21·7 % (*n* 5) normal weight BMI, 52·2 % (*n* 12) overweight BMI and 21·7 % (*n* 5) had an obese BMI.

Following this, gestational characteristics were assessed as shown in Table 2. Of the women presenting for antenatal care, the mean ± sd gestation was 12·4 ± 1·4 weeks’ gestation, whereby 69·8 % (*n* 229) of the pregnancies were planned. Additionally, on average (mean ± sd) women reported finding out they were pregnant at 4·4 weeks ± 2·3 weeks gestation.

**Table 2:** Gestational characteristics

	*n*	%
Number of weeks gestation (weeks)		
Mean	12·4	
sd	1·4	
Found out I was pregnant (weeks)		
Mean	4·4	
sd	2·3	
Planned pregnancy		
Yes	229	69·8
No	97	29·6
No answer	2	0·6
Number of pregnancies		
Primigravida	110	33·5
Multigravida	192	58·6
Grand-multigravida (≤5)	26	7·9
Parity		
Nulliparous	130	39·6
Primiparous	99	30·2
Multiparous	92	28·0
Grand-multiparous (≤5)	7	2·1
Last pregnancy		
1–2 years	111	33·8
3–4 years	45	13·7
4–5 years	16	4·9
>5 years	49	14·9
No answer	107	32·6

Moreover, results showed that 33·5 % (*n* 110) of women were experiencing their first pregnancy. In contrast, over 66·3 % (*n* 218) of women had experienced ≥1 pregnancy before. Over half of women had previously given birth to at least one live child before. In fact, it was noted that 33·8 % (*n* 111) had experienced a previous pregnancy within 1–2 years prior to this antenatal visit.

### Folic acid supplementation practices

Folic acid supplementation practice analysis is shown in Table 3. The majority of this cohort were taking folic acid daily. For 30·5 % (*n* 100) of women, folic acid supplementation started >12 weeks before pregnancy. Whereas 61·0 % (*n* 199) of women reported starting supplementing when they found out they were pregnant. The remaining 4·0 % (*n* 13) failed to answer and 4·6 % (*n* 15) stated other answers such as *‘I started once my GP advised me’, ‘I am about to start’, ‘Just before I found out’, ‘Week 8 of pregnancy’ and ‘7 to 8 weeks before becoming pregnant’*. Of those who participated, 46·6 % (*n* 153) decided themselves and 36·3 % (*n* 199) were informed by their doctor to start folic acid supplementation.

**Table 3:** Folic acid supplementation practices

	*n*	%
Taking FA		
Yes	315	96
No	13	4
FA compliance		
Every day	314	95·7
Once a week	4	1·2
No answer	10	3·0
Commencement of FA		
>12 weeks before pregnancy	100	30·5
When I found out I was pregnant	200	61·0
Other	15	4·6
No answer	13	4·0
Advice on FA consumption		
Doctor/nurse	119	36·3
Family member/friend	28	8·5
I decided myself	153	46·6
Other	20	6·1
No answer	8	2·4
FA dose		
400 μg	216	65·9
5 mg	22	6·7
Other	2	0·6
Dose not specified	85	26·0
Not taking FA	3	0·9
Meeting dose requirements		
Met requirements	190	57·9
Did not meet requirements	50	15·2
Unknown	88	26·8

A total of twenty-nine folic acid brands were reported by women. While 29 % (*n* 95*)* of women failed to answer. Overall, 65·9 % (*n* 216) of women were taking 400 μg and 6·7 % (*n* 22) were taking the higher dose of 5 mg. Of those who answered, it was determined that 57·9 % (*n* 190) met their folic acid dose requirements and 15·2 % (*n* 50) did not.

Further analysis on reaching folic acid requirements for BMI was carried out on those who reported to be taking folic acid as highlighted in Table 4. Results showed those women with a healthy BMI *n* 185, 99·5 % (*n* 184) met folic acid dose requirements and 0·5 % (*n* 1) did not. Whereas those classified as obese *n* 55, only 10·9 % (*n* 6) met their dose requirements and 89·1 % (*n* 49) did not. Women with obesity were less likely to meet their folic acid dose requirements in comparison with women without obesity. Fisher’s exact tests rendered this significant (*P =<* 0·001).

**Table 4:** Meeting folic acid (FA) requirements according to BMI

Meeting FA requirements		Not obese	Obese	Fishers exact test (exact sig two-sided)
FA requirements met	Count	184	6	<0·001
	% within BMI group	99·5	10·9	
Did not meet FA requirements	Count	1	49	
	% within BMI group	0·5	89·1	
	Total	185	55	

## Discussion

This study found that the majority of women presenting for antenatal care were complying to national standards by supplementing with folic acid daily. However, 30·5 % (*n* 100) complied with public health recommendations commencing supplementation before 12 weeks prior to conception. In addition, within the 96 % (*n* 315) of women who reported to be taking folic acid at present, the timing of such supplementation varied from any time between >12 weeks prior to conception up until the day of their first antenatal booking appointment. To put this into context, women presenting at their first appointment ranged from 4 to 30 weeks’ gestation.

In total, one-third of mothers commenced supplementation within the recommended time frame. This predisposed 65·6 % (*n* 215) of women, to an increased risk of having RBC folate levels low enough to cause an NTD as the duration of supplementation was considered too short^(17,18)^. Such findings are consistent with previous research^(5,44–47)^. It is clear that in spite of recommendations, Irish women are failing to comply to folic acid supplementation guidelines^(2)^. Evidence has shown that only 27–44 % of women are commencing supplementation prior to conception^(44–47)^. Studies have also commented on the duration of preconception supplementation, where results have shown that no more than 25–36 % of women are taking folic acid for the recommended length of 12 weeks prior to conception^(5,44)^. It is also known that the majority of women commence folic acid supplementation when they find out they are pregnant, which is generally 4–8 weeks after their last menstrual period^(46,48)^. This is commonly due to the fact that a high percentage of pregnancies worldwide are unplanned resulting in delayed supplementation practices^(44,45)^. In this current study, 60·1 % (*n* 200) of mothers started supplementation when they found out they were pregnant which on average was 4·4 ± 2·3 weeks into their first trimester of pregnancy. Commencement of folic acid supplementation at this stage just before or after neural tube closure is likely to have been ineffective in the prevention of NTD^(2)^. Consequently, women are not supplementing within the most crucial time period when increased folic acid is required due to high rates of cell division. Potentially, malformations of the neural tube may have already occurred before these women even knew that they were pregnant. These findings are of clear clinical relevance, as essentially a high percentage of women within this study did not achieve optimal protection against NTD despite the fact it could have been prevented. Action is needed to address this problem to ensure that women initiate folic acid supplementation at the correct time point of pregnancy. To do so, increased and improved public health messages targeting the importance and benefits of appropriate timing of supplementation need to be developed and promoted to improve adherence. Such initiatives could be introduced within primary care settings or in antenatal classes to promote folic acid supplementation. This could potentially allow those who are not pregnant to be aware of the recommended guidelines prior to conception and would also enable those who are currently pregnant to be educated in advance of a possible second pregnancy.

Analysis of folic acid supplementation dosage found that majority of women within this study were supplementing with the standard folic acid dose of 400 μg. 6·7 % (*n* 22) women were taking the higher dose of 5 mg, out of the 78 (23·8 %) women who presented with obesity based on ethnic specific BMI classifications. This finding indicated that an insufficient amount of women with obesity were supplementing appropriately. Thus, it was not surprising that those with obesity were less likely to meet folic acid supplementation dose requirements (*P =* 0·001) and that the success rate of women with obesity reaching their dose requirements was extremely low. Alarmingly, 89·1 % (*n* 55) of women with obesity did not meet their dose requirements.

The issue of inappropriate folic acid dose supplementation amongst women with obesity is clearly an advancing problem; however, research surrounding this topic remains sparse. To our knowledge, Cawley *et al.* (2015) and O’Malley *et al.* (2018) were two of the first to investigate this topic whereby high-dose folic acid uptake in women with obesity was proven to be worryingly low^(5,26)^. Their findings saw only 5·7 % and 9·5 % of women with obesity to be taking the higher 5 mg dose of folic acid, respectively^(5,26)^. This is consistent with our findings, as low rates of women with obesity were supplementing with the appropriate high dose of folic acid. It may be considered that as nearly half of the women decided to start supplementation themselves in this study, they lacked knowledge and awareness of the different folic acid doses available for women with and without obesity. Also, by deciding themselves this prevented women with obesity from being exposed to the possibility of being prescribed the higher dose of folic acid from their doctor. Thus, the requirement of high-dose folic acid prescription may actually be posing as a barrier to its use as some women may be unable to avail of GP services due to specific constraints, i.e. cost. For those who did seek advice from their doctor it remain unknown if the higher dose was prescribed. What is more, as it is common that women typically do not present within the early stage of pregnancy, this prevents the opportunity for medical intervention to arise. It is evident that more research is needed to specifically assess why high-dose folic acid compliance in women with obesity is so poor^(5)^.

Although research regarding folic acid supplementation dose remains sparse, multiple studies have investigated the factors associated with preconceptual folic acid supplementation. Research has indicated that having a higher socio-economic status, being married, a non-smoker, having an increased maternal age and low parity to be important predictors of preconceptual folic acid supplementation^(49–51)^. While, alternatively, a lower socio-economic status, younger age, smoker, low educational attainment, high parity and being of non-Caucasian ethnicity have each been identified as detriments towards folic acid supplementation^(46,49–52)^.

Taking these identified confounding factors into consideration, it is interesting to first note that this study’s cohort reported to have high educational attainment. This may be reflected in why a high percentage of women chose to start folic acid supplementation themselves without being advised from their doctor. Furthermore, it may be considered that this cohorts high parity influenced their supplementation practice as it is known that women who have experienced pregnancy become complacent with supplementation recommendations^(44)^. This is of high concern considering that higher parity is also associated with an increased risk of NTD^(53)^. To add to this, high parity is linked with poor pregnancy planning^(54,55)^. As less than half of pregnancies are planned worldwide, having a planned pregnancy has been shown to be a consistent factor in predicting periconceptional folic acid supplementation use^(2,45,51)^. Interestingly, this study contradicts such evidence as although over half of this study cohort reported planning their pregnancy, they still failed to supplement pre-conceptually.

Furthermore, having an obese BMI can be recognised as a confounding factor towards supplemental practices. Overall, folic acid supplementation has been shown to be lower in women with obesity, and they can be considered less likely to take preconceptual folic acid than those of a normal-weight BMI^(56)^. Research has also indicated for unplanned pregnancies to be higher in women with obesity, and they can be considered less likely to receive folate through their diet^(56,57)^. Thus, as nearly one quarter of women attending their first antenatal appointment within this study were obese, it can be said that obesity may have been one of the main confounding factors towards inadequate supplementation. These inadequacies along with obesity are of high concern as it may be expected that this problem will continue to escalate within the coming years due to the ongoing rise of obesity among women of a childbearing age, aged 18–37 years in Ireland today^(58)^. In fact, it could be hypothesised that if obesity rates continue to rise and current supplementation practices remain suboptimal, the problem of NTDs will only continue to worsen. Subsequently this could lead to numerous adverse outcomes for women, families and healthcare systems. Considering such points, public health strategies evidently need to be more mindful of these confounding factors towards folic acid supplementation when planning approaches to prevent complacency and to ensure that awareness and education are increased amongst all women particularly those within such vulnerable groups.

This study has many strengths, unlike other studies, ethnicity was specifically accounted for. As evidenced by Prentice and Jebb (2001), BMI has limitations when comparing ethnic groups with distinctively different body proportions or physiques^(59)^. It has been shown that the maternal composition of Indian women is different to that of Irish women, as they have higher total body and visceral fat percentages^(28)^. This predisposes women of an Asian descent to having a higher BMI and increased pregnancy related complications^(28)^. Thus, different BMI cut-off points have been proposed for this ethnic group of which were used within this study. It is clear that Asian BMI cut-off points need to be further recognised within future studies due to the known variability of maternal BMI^(28)^. What is more, with the ongoing rise of Ireland’s multicultural population, particular attention needs to be made to ensure that public health approaches surrounding folic acid supplementation are suitable for all groups of women from different ethnic backgrounds. This is particularly important given the known fact that being non-Caucasian is a detriment towards folic acid supplementation^(52)^.

Second, folic acid supplementation practices were assessed using a structured questionnaire. Third, anthropometric measurements were standardised and consecutively performed by a trained researcher. This allowed for BMI to be calculated based on accurate measurements of weight and height and not self-reporting. Previous research has been based on self-reporting and pre-pregnancy measurements causing the reliability of these studies to be questioned^(60,61)^. In particular, studies have shown for self-reported weight and height to be inaccurate in both obese and female subjects^(56)^. Hence, the reliability of anthropometric measurements and BMI calculations within this study was enhanced by the method employed. Lastly, as ethnicity was controlled for, this too prevented the misclassification of BMI.

A potential weakness lies in this study’s method employed for the completion of participant questionnaires. As questionnaires were self-reported, supervision was not provided subjecting responses to recall bias. In particular, the mother’s retrospective recollection of the timing of preconceptual folic acid supplementation might be at risk of bias. Additionally, social desirability may have impacted participants responses to certain questions as it is known that some women may overestimate their compliance with public health recommendations during pregnancy^(62)^. This potentially may have caused for the actual prevalence of folic acid supplementation compliance reported to be inaccurate^(62)^. However, as stated by Tarrant *et al.* (2011), such inaccuracies are unlikely to differ from other similarly designed studies^(45)^.

Due to the limited time frame of the current study, the questionnaire used was not piloted causing the validity of this assessment tool to be questioned. Also, the direct relationship between maternal characteristics and folic acid supplementation practices was not investigated. Hence, the confounding factors towards this cohort’s suboptimal folic acid supplementation practices are questionable and remain uncertain. It must also be stated that participants overall risk towards NTD was not fully determined as dietary folate intake and maternal serum folate levels were not investigated.

Furthermore, as this was a single-sited cross-sectional study capturing only women within the south-west of Ireland, the sample cohort was small and characteristics may only represent a minority. In addition, although BMI and ethnicity were controlled for, no data were collected on how long the women of an Asian descent had been living in Ireland. This is significant as the Irish lifestyle may have altered their adiposity and BMI classifications given the length of their time living here in Ireland^(28)^. To add to this, although BMI is an inexpensive and practical form of measurement, it does have its limitations. As BMI is a surrogate marker and it does not measure adipose tissue directly preventing information on fat distribution to be gathered and interpreted^(31,59)^.

The work presented in the current study shows that voluntary fortification with supplementation of women prenatally is not an effective strategy to increase folic acid intake amongst women prenatally. Women of childbearing age do not always plan pregnancies, and therefore folic acid supplementation may not be a factor that they are considering. In order to reduce rates of the occurrance of NTD folate levels need to be optimised pre-conceptionally and up till 21–28 d gestation when the neaural tube fuses. Where the rates of NTD in Ireland is at 9 in 10 000, this could be reduced to 5–6 cases in 10 000 with the introduction of mandatory fortification^(63)^. In the USA, NTD rates dropped with the introduction of mandatory folic acid fortification, and these rates were maintained and remained stable a decade on^(64)^. Concerns of the previously speculated link between folic acid supplementation and increased incidences of certain cancers have now been superceded by new data looking at outcomes long after the introduction of mandatory fortification in other countries. Despite an initial increase in colorectal cancers followed by a downwards trend, studies in the USA propose fortification programs may be responsible for the long-term downward trend of colorectal cancers in the USA^(65)^. Seven years later, a prospective study looking at over 86 K women post introduction of manditory fortification confirmed that folate intake both from total and synthetic forms was associated with a lower risk of overall colorectal cancer after a long latency period^(66)^. Similarly, a study conducted in Australia evaluating colorectal cancer incidence patterns before and after introduction of mandatory folic acid fortification found no evidence that the incidence had been influenced^(67)^. These prospective studies are evidence that mandatory fortification of staple foods with folic acid to reduce NTD are not only effective at lowering rates of NTD but are also safe strategies to implement at a population level. With a reported 59 294 births in Ireland in 2019^(68)^, introduction of mandatory fortification of staple foods with folic acid has the potential to reduce births with NTD from 53 to 29–35 *per anum*, based on rates of 5–6 per 10 000 achieved in countries which have implemented such measures^(63)^. At an estimated cost of half a million during the lifetime of a patient born with a *spina bifida*^(69)^ this could present an economic cost saving of 14·5–17·5 million each year. These direct costs do not even acount for the burden of disease to the affected patient and family.

## Conclusion

To conclude, the folic acid supplementation practices by mothers within this study were suboptimal to prevent their risk of having a pregnancy resulting in an NTD. Findings indicate that a large proportion of women are not complying with national folic acid supplementation guidelines as they are failing to commence supplementation at the correct time. Despite the numerous efforts of health promotion, raised awareness and educational strategies already implemented to date, folic acid supplementation practices remain poor. It is evident that public health approaches need to be improved and adapted in order to optimise folic acid supplementation in women and to initiate a behaviour change. The message that women of childbearing age should be supplementing with folic acid regardless as to whether they are trying to conceive or not needs to be emphasised. In fact, women from a young age should be targeted with folic acid supplementation education with the intention of preparing them for childbearing in the future.

It is also important to note that primary prevention of NTD by periconceptual folic acid supplementation is also a major public health opportunity for all multi-disciplinary care teams. Our findings highlight the need for increased education and awareness of supplementation among not only women themselves but also amongst health care professionals to ensure that folic acid supplementation compliance, commencement and particularly dose recommendations are implemented in line with best practice guidelines to date.

Lastly, the current study highlights women most at risk of non-compliance with supplementation recommendations. Women with obesity who are already predisposed to having a pregnancy affected by NTD are not taking the appropriate steps to combat this risk. Folic acid supplementation practices within this group are suboptimal on a number of counts. Particularly this study brings to light that women are not supplementing with the required and appropriate higher dose of folic acid needed to prevent NTD in pregnancy. With the high and rising rate of obesity, it can be concluded that unless appropriate supplementation is addressed and resolved within this group of women, the clinical issue of NTD will continue to grow.

Taking these points into consideration, it would be first recommended to adjust education and communication approaches to suit young women, i.e. approaches should include using social media or television platforms. Future folic acid supplementation strategies also need to be cognisant of the changing maternal population and should target women with obesity and those within multicultural groups. Furthermore, increased promotion and education of folic acid supplementation not only needs to be directly delivered to women of child-bearing age but also to health care professionals in the primary care setting to ensure the prescription of higher dose folic acid is being implemented.

Lastly, considering the findings from this study and in view of the positive impact on NTD incidence with no adverse effects reported in countries with long established mandatory fortification of staple foods with folic acid, is it time Ireland adopted such an approach to this public health problem?

## Supplementary material

For supplementary material accompanying this paper visit https://doi.org/10.1017/S1368980022001574. click here to view supplementary material
