# Supplementary material for: An evaluation of adherence to folic acid supplementation in pregnant women during early gestation for the prevention of neural tube defects
Source: Public Health Nutr. 2022 Jul 25;25(11):3025–35. doi: 10.1017/S1368980022001574 (PMC9991708; doi:10.1017/S1368980022001574)
Supplement: Supplementary file 1 [file S1368980022001574sup.zip › S1368980022001574sup002.docx]

**
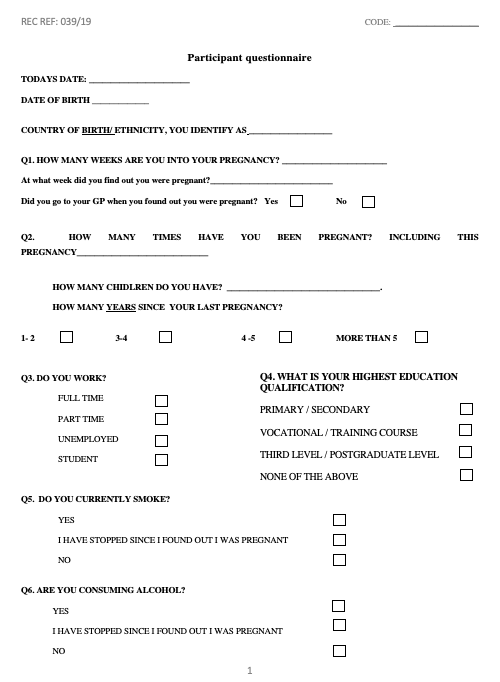
Supplementary Material 1.** Participant Questionnaire Page 1.

**Supplementary Material 1:** Participant Questionnaire Page 2.

**
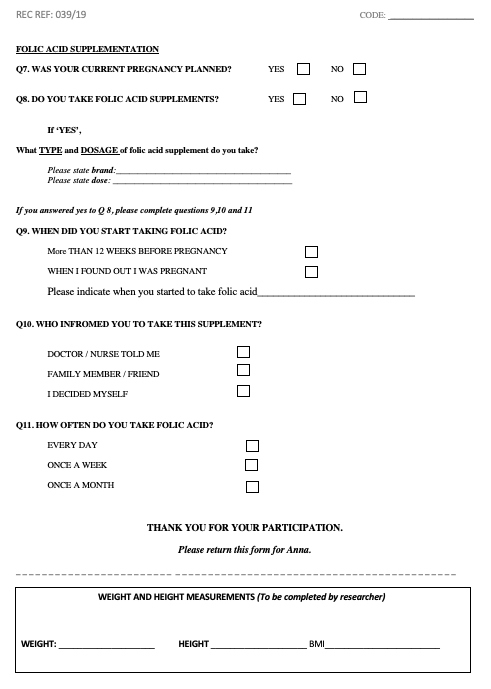
**
